# Persistent Hyperinsulinemic Hypoglycemia After Insulinoma Surgery: Diagnostic Impact of GLP‐1 Receptor PET/CT After Negative Conventional Imaging

**DOI:** 10.1155/crie/7874466

**Published:** 2026-10-08

**Authors:** Lorenzo Taglia, Alessandra Guercio, Silvia Migliari, Angela Vazzana, Riccardo C. Bonadonna, Livia Ruffini, Alessandra Dei Cas

**Affiliations:** ^1^ Department of Medicine and Surgery University of Parma Parma Italy unipr.it; ^2^ Nuclear Medicine Division University Hospital of Parma Parma Italy parmahospital.org; ^3^ Endocrinology and Metabolic Diseases Azienda Ospedaliero-Universitaria of Parma Parma Italy unipr.it; ^4^ Endocrinology, Diabetes and Metabolism Department of Medicine University and Hospital Trust of Verona Verona Italy ospedaleuniverona.it

**Keywords:** ^68^Ga-NODAGA-Exendin-4, endoscopic ultrasound, GLP-1 receptor PET/CT, insulinoma, radiofrequency ablation, recurrent hypoglycemia

## Abstract

**Background:**

Localization of recurrent or residual insulinoma may be particularly challenging in postoperative setting, especially in the presence of postoperative anatomical distortion and inconclusive conventional imaging.

**Case Presentation:**

A 71‐year‐old man developed recurrent endogenous hyperinsulinemic hypoglycemia following apparently curative surgical enucleation of a pancreatic insulinoma. The postoperative course was complicated by intra‐abdominal hemorrhage and pseudocyst formation, resulting in marked anatomical distortion. Despite persistent biochemical evidence of insulinoma recurrence, serial contrast–enhanced computed tomography (CT) and ^68^Ga‐DOTATOC PET/CT failed to identify residual disease. Subsequent glucagon‐like peptide‐1 receptor (GLP‐1R) imaging with ^68^Ga‐NODAGA‐Exendin‐4 PET/CT demonstrated focal tracer uptake in the pancreatic head, highly suggestive of recurrent or residual insulinoma. Endoscopic ultrasound (EUS) subsequently confirmed a small lesion in the uncinate process, which was successfully treated with EUS–guided radiofrequency ablation (EUS‐RFA). Following the procedure, hypoglycemic episodes resolved, with normalization of glucose, insulin, and C‐peptide levels and no signs of recurrence at follow‐up.

**Conclusion:**

This case highlights the clinical value of GLP‐1 receptor PET/CT for localizing insulinoma in the context of negative conventional or somatostatin receptor (SSTR)–based imaging. Accurate functional localization may directly guide minimally invasive therapeutic interventions and help avoid high‐risk reoperative pancreatic surgery.

## 1. Introduction

Insulinomas are the most common functioning pancreatic neuroendocrine tumors (pNETs), although they remain rare, with an estimated incidence of 1–4 cases per million individuals per year [1]. Surgical resection is the treatment of choice and is curative in most patients [2]. However, localization of recurrent or residual disease may be particularly challenging, especially in the presence of postoperative anatomical distortion or when lesions are small and difficult to detect [2].

Conventional imaging techniques, including contrast‐enhanced computed tomography (CT), magnetic resonance imaging (MRI), and endoscopic ultrasound (EUS), are routinely used for tumor localization, but small lesions may remain undetected. In addition, the diagnostic performance of somatostatin receptor (SSTR)–based PET/CT may also show limited sensitivity because insulinomas frequently exhibit variable SSTR expression [3–5].

Glucagon‐like peptide‐1 receptor (GLP‐1R) PET/CT imaging with radiolabeled exendin analogs has emerged as a highly sensitive technique for insulinoma detection, owing to the high density of GLP‐1R expressed by most benign insulinomas. In this context, the Radiopharmacy Lab of the University Hospital of Parma has validated the in‐house radiosynthesis of ^68^Ga‐NODAGA‐Exendin‐4 [6], as described in the Supporting Information.

Accurate functional localization is increasingly important, as it may facilitate targeted therapeutic strategies, including minimally invasive approaches such as EUS‐guided radiofrequency ablation (EUS‐RFA) [7].

We report a case of recurrent endogenous hyperinsulinemic hypoglycemia following previous surgical enucleation of an insulinoma, in which conventional imaging and SSTR‐based PET/CT were nondiagnostic. GLP‐1R imaging with ^68^Ga‐NODAGA‐Exendin‐4 PET/CT enabled precise lesion localization and directly guided successful EUS‐RFA, thereby avoiding new pancreatic surgery.

## 2. Case Presentation

A 71‐year‐old man with a history of atrial fibrillation and bicameral pacemaker implantation presented with recurrent episodes of tremor, diaphoresis, and confusion beginning in June 2023, which were relieved by oral glucose intake. Laboratory evaluation showed a fasting plasma glucose of 44 mg/dL, insulin 4 µU/mL, and elevated chromogranin A levels (576.6 ng/mL). A subsequent supervised short fasting test confirmed endogenous hyperinsulinemic hypoglycemia: after 17 h of fasting, the plasma glucose level was 51 mg/dL, insulin was 6 µU/mL, and C‐peptide was 0.77 nmol/L. Diazoxide therapy was then initiated with partial symptomatic control.

Contrast‐enhanced abdominal CT identified a 10 × 10 mm hypervascular nodule in the pancreatic head. In August 2023, a ^68^Ga‐DOTATOC PET/CT showed intense SSTR (SSTR2 and SSTR5) expression in the same region (Figure 1a).

**Figure 1:**
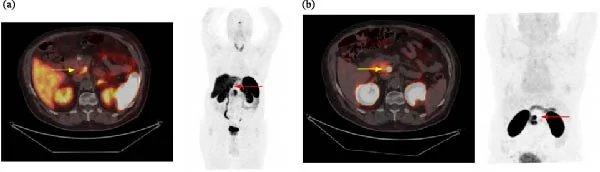
Comparison between (a) August 2023 ^68^Ga‐DOTATOC and (b) September 2023 ^68^Ga‐NODAGA‐Exendin‐4 fused PET/CT axial section and MIP.

To further characterize the lesion, a ^68^Ga‐NODAGA‐Exendin‐4 PET/CT was performed in September 2023, confirming a single lesion with marked GLP‐1R overexpression (Figure 1b).

^68^Ga‐DOTATOC and ^68^Ga‐NODAGA‐Exendin‐4 radiosynthesis results, fully compliant with established specifications, are summarized in Supporting Information.

### 2.1. Diagnostic Assessment

The combination of hypoglycemia, endogenous hyperinsulinemia, and concordant functional imaging findings was highly suggestive of insulinoma. Biochemical preoperative evaluation of pituitary, adrenal, and parathyroid function excluded multiple endocrine neoplasia type 1 (MEN1).

In October 2023, robotic‐assisted surgical enucleation was attempted but required conversion to open procedure due to intraoperative difficulties in lesion localization despite intraoperative ultrasound and indocyanine green guidance.

Histopathological examination revealed a well‐differentiated neuroendocrine tumor (NET G1), measuring 1.7 × 1.4 × 1.2 cm, with Ki‐67 expression < 2%. Immunohistochemistry was positive for chromogranin A and CD56, in addition to diffuse insulin positivity, confirming insulinoma.

Surgical margins were reported as negative, and diazoxide therapy was discontinued.

The postoperative course was complicated by a large intra‐abdominal hemorrhage: few weeks after hospital discharge, the patient was admitted to the emergency department for severe abdominal pain, and emergency CT showed a rapidly expanding hematoma of 9 × 8 × 11.5 cm with active arterial bleeding. Selective embolization of the right gastroepiploic artery was successfully performed. Over subsequent weeks, the hematoma evolved into a large pseudocyst (10 × 12 cm), causing compression of adjacent structures. Endoscopic drainage was attempted but was not feasible due to altered anatomy.

Two months after surgery, hypoglycemic symptoms recurred. Between January and June 2024, repeated fasting tests documented recurrent hypoglycemias with inappropriately elevated insulin (glucose 51–75 mg/dL and insulin 5.6–6 µU/mL), leading to prompt reintroduction of the diazoxide therapy. Serial CT studies throughout 2024 showed gradual regression of the pseudocyst with persistent fibrotic changes, and by September 2024, the hematoma had nearly been resolved, revealing only residual fibrotic tissue without evidence of recurrence of insulinoma.

In October 2024, a repeat ^68^Ga‐DOTATOC PET/CT failed to identify pathological pancreatic uptake and revealed only focal tracer accumulation in the thyroid isthmus (Figure 2a). Fine needle aspiration cytology classified the thyroid lesion as Bethesda category II [8], and calcitonin measurement in the needle washout was negative, supporting a benign diagnosis.

**Figure 2:**
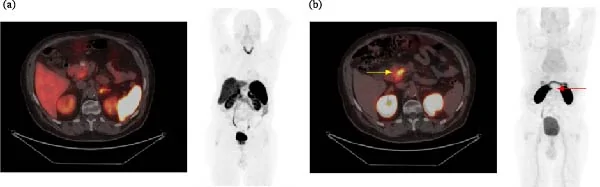
Comparison between (a) October 2024 ^68^Ga‐DOTATOC and (b) November 2024 ^68^Ga‐NODAGA‐Exendin‐4 fused PET/CT axial section and MIP.

Given the persistence of hypoglycemic symptoms despite negative conventional and SSTR‐based imaging ^68^Ga‐DOTATOC PET/CT, a repeat PET/CT with ^68^Ga‐NODAGA‐Exendin‐4 was performed in November 2024. Radiosynthesis details are reported in Supporting Information.

Notably, PET/CT with ^68^Ga‐NODAGA‐Exendin‐4 identified two new focal areas of intense tracer GLP‐1R uptake posterior to surgical clips in the pancreatic head, highly suspicious for small residual or recurrent insulinoma (Figure 2b), although interpretation was partially limited by postoperative anatomical distortion. At that time, the fasting glucose level was 61 mg/dL, insulin 13 µU/mL, and C‐peptide 0.62 nmol/L. In March 2025, EUS could visualize a 5–6 mm solid, well‐demarcated hypoechoic lesion in the uncinate process of the pancreas. The EUS‐FNA revealed clusters of neuroendocrine cells, consistent with low‐grade NET. Although FNA could not reliably distinguish between hyperplastic islets and a residual tumor tissue, the clinical context of the combination of recurrent hyperinsulinemia—together with the positive ^68^Ga‐NODAGA‐Exendin‐4 PET/CT—supported the diagnosis of residual insulinoma.

### 2.2. Treatment

Repeat pancreatic surgery was considered technically challenging and potentially high‐risk because of the patient’s frailty and the substantial postoperative anatomical distortion resulting from previous complications. Given the small lesion size and its accessibility by EUS, EUS‐RFA was selected as a minimally invasive and potentially curative therapeutic strategy.

In May 2025 the lesion was successfully ablated under EUS guidance without procedural complications, and diazoxide therapy was discontinued immediately thereafter.

### 2.3. Outcome and Follow‐Up

Following ablation, capillary glucose monitoring showed a complete resolution of hypoglycemia. In June 2025, fasting glucose was 81 mg/dL, HbA1c was 33 mmol/mol, and insulin and C‐peptide levels had normalized.

At follow‐up in late 2025, the patient remained asymptomatic, with stable glycemic control and no recurrence of hypoglycemic episodes. A new ^68^Ga‐DOTATOC confirmed the previously detected isolated thyroid uptake. Despite normal calcitonin levels, the thyroid nodule underwent a new FNAC exam, which confirmed a benign lesion.

Of note, as reported by Bentestuen et al. [9], incidental thyroid uptake is a recognized finding on SSTR PET/CT, reported in ~4%–11% of scans performed in patients with known or suspected neuroendocrine tumors. In most cases, particularly when diffuse, this uptake is not related to malignancy but reflects benign conditions such as inflammatory or leukocytic infiltration. In our patient, subsequent cytological evaluation confirmed the benign nature of the lesion, supporting this interpretation.

Clinically, the patient remained asymptomatic with stable weight and complete resolution of neuroglycopenic and adrenergic symptoms. A summary of the patient’s biochemical parameters over the clinical course is provided in Table 1.

**Table 1:** Summary of biochemical parameters throughout the clinical course.

Clinical time point	Plasma glucose (mg/dL)	Serum insulin (µU/mL)	C‐peptide (nmol/L)	Glycated hemoglobin (mmol/mol)
At presentation	44	4	0.75	22
After surgery	64	4	0.45	22
At biochemical recurrence	61	13	0.62	28
Long follow‐up after EUS‐RFA	81	8	1.49	33

Abbreviation: EUS‐RFA, endoscopic ultrasound‐guided radiofrequency ablation.

## 3. Discussion

Accurate localization of insulinomas remains crucial, as complete surgical resection is curative in the vast majority of patients. Conventional imaging with contrast‐enhanced CT and MRI is generally used as the initial approach, although sensitivity may be limited, particularly for small lesions. A meta‐analysis reported pooled sensitivities of 54% for both CT and MRI, with specificities of 75% and 65%, respectively [10]. EUS provides higher diagnostic performance, with a sensitivity of 81% and a specificity of 90% [11].

Tumor localization becomes particularly challenging when conventional imaging is negative or inconclusive, as may occur in patients with recurrent endogenous hyperinsulinemic hypoglycemia after apparently curative surgery [12]. In this setting, functional imaging may provide additional diagnostic information.

Although SSTR‐based PET/CT with radiolabeled analogs such as ^68^Ga‐DOTATOC is widely used for the evaluation of neuroendocrine tumors, its performance in insulinoma is limited by heterogeneous and often low SSTR expression [12–14]. Accordingly, a meta‐analysis [10] reported a pooled sensitivity of only 39% for SSTR‐based PET/CT, underscoring its limited sensitivity for insulinoma localization.

In contrast, GLP‐1 receptor imaging with ^68^Ga‐NODAGA‐Exendin‐4 PET/CT selectively targets receptors that are highly expressed in most benign insulinomas and has emerged as one of the most effective techniques for tumor localization [12, 15]. A systematic review and meta‐analysis reported a pooled sensitivity of 94% for Exendin‐4 PET/CT [16], supporting its high diagnostic performance, particularly in localized indolent insulinomas, in which GLP‐1 receptor expression is typically preserved. Importantly, GLP‐1 receptor–targeted PET/CT may provide substantial diagnostic value in patients with negative or inconclusive conventional imaging, in whom insulinoma localization remains particularly difficult [16, 17].

The present case highlights the diagnostic challenges associated with persistent endogenous hyperinsulinemic hypoglycemia following apparently curative insulinoma surgery. Despite histologically complete resection and initially concordant imaging findings, the patient developed biochemical and clinical recurrence, indicating the presence of residual disease that remained occult on conventional imaging.

Postoperative hemorrhage and pseudocyst formation resulted in marked distortion of pancreatic anatomy, substantially limiting the sensitivity of repeated CT examinations. In this context, ^68^Ga‐DOTATOC PET/CT became negative during follow‐up, whereas ^68^Ga‐NODAGA‐Exendin‐4 PET/CT identified focal GLP‐1R uptake within the surgical field. This discordance is consistent with the known heterogeneity of SSTR expression in insulinomas and supports the value of GLP‐1R–targeted imaging in well‐differentiated disease [13].

Importantly, GLP‐1R PET/CT findings directly influenced clinical management. Whole‐body imaging excluded multifocal or metastatic disease, supporting a localized therapeutic approach. EUS subsequently confirmed a small lesion in the uncinate process, and EUS‐RFA was successfully performed, avoiding high‐risk reoperation in a patient with complex postoperative anatomy [18].

This case adds to the growing evidence supporting the clinical utility of GLP‐1R PET/CT in patients with persistent endogenous hyperinsulinemic hypoglycemia and inconclusive conventional or SSTR‐based imaging. Moreover, it highlights the potential role of GLP‐1R imaging in guiding personalized, minimally invasive treatment strategies when repeat surgery is considered undesirable or high risk. Importantly, our center currently represents the only Italian institution with an active clinical and translational program in GLP‐1R PET/CT imaging, allowing direct integration of this emerging modality into routine endocrine practice.

While accumulating evidence suggests excellent diagnostic performance, the precise placement of GLP‐1R PET/CT within the diagnostic algorithm and its influence on patient management still require validation in larger prospective studies.

Future multicenter investigations will be essential to establish standardized indications and define its role alongside conventional and SSTR‐based imaging techniques [19–21].

Overall, this case highlights the potential clinical utility of GLP‐1R PET/CT in patients with persistent endogenous hyperinsulinemic hypoglycemia and inconclusive conventional or SSTR‐based imaging after insulinoma surgery.

## Funding

The authors declare no public or commercial funding. Open access publishing facilitated by Universita degli Studi di Parma, as part of the Wiley ‐ CRUI‐CARE agreement.

## Disclosure

The authors have nothing to report.

## Ethics Statement

This study was conducted in accordance with the institutional review board of Comitato Etico Territoriale Area Vasta Emilia Nord (Prot. Number 19585 of April 30, 2026).

## Consent

Written informed consent was obtained from the patient for the use of clinical and image data, including publication of deidentified clinical details and laboratory findings.

## Conflicts of Interest

The authors declare no conflicts of interest.

## Supporting Information

Additional supporting information can be found online in the Supporting Information section.

## Supporting information


**Supporting Information** The Supporting Information file provides additional technical information on the radiosynthesis of 68Ga‐DOTATOC and 68Ga‐NODAGA‐Exendin‐4. It includes radiosynthesis and quality control results, HPLC and TLC chromatograms from the first PET/CTs, and production data of both radiopharmaceuticals.

## Data Availability

The data underlying this article are not publicly available due to patient privacy but are available from the corresponding author upon reasonable request.
