# Supplementary material for: Persistent Hyperinsulinemic Hypoglycemia After Insulinoma Surgery: Diagnostic Impact of GLP‐1 Receptor PET/CT After Negative Conventional Imaging
Source: Case Rep Endocrinol. 2026 Oct 8;2026:7874466. doi: 10.1155/crie/7874466 (PMC13647209; doi:10.1155/crie/7874466)
Supplement: Supplementary file 1 [file CRIE-2026-7874466-s001.docx]

Both ^68^Ga-DOTATOC and ^68^Ga-NODAGA-Exendin-4 were synthesized using an automated module and a GMP-certified ^68^Ge/^68^Ga generator, ensuring reproducibility and clinical-grade quality. Radiochemical purity was consistently high, typically above 97% by HPLC and confirmed at 100% by TLC, which demonstrates the robustness of the process. Regarding ^68^Ga-NODAGA-Exendin-4 the injected peptide amounts are kept in the microgram range, well below pharmacological thresholds, while maintaining sufficient activity for high-quality PET imaging.

^68^Ga-DOTATOC radiosynthesis results, fully compliant with established specifications, are summarized in the Table (a). HPLC and TLC chromatograms of the first PET/TC (August 2023) are shown in the Figure (a).

^68^Ga-NODAGA-Exendin-4 radiosynthesis results, fully compliant with established specifications, are summarized in the Table (b). HPLC and TLC chromatograms of the first PET/TC (September 2023) are shown in the Figure (b).

From a radiopharmaceutical perspective, this case underscores the reliability of in-house synthesized ^68^Ga-labelled peptides under GMP conditions. The consistent radiochemical purity, low peptide mass, and reproducibility across multiple syntheses ensured high-quality imaging and supported the use of GLP-1R PET/CT in longitudinal patient management.

The reproducibility of radiochemical purity, injected peptide mass, and administered activity across multiple productions, including follow-up scans, ensured consistent image quality and supported the use of these tracers for longitudinal patient monitoring.

| a. **^68^Ga-DOTATOC synthesis report** | | | | |
| --- | --- | --- | --- | --- |
|  | **RCY** | **RCP (HPLC)** | **RCP (TLC)** | **Administered dose** |
| ***n. 1*** | 62.47% | 99.64% | 100% | 122 MBq |
| ***n. 2*** | 62.41% | 99.46% | 100% | 101 MBq |

| b. **^68^Ga-NODAGA-Exendin-4 synthesis report** | | | | | |
| --- | --- | --- | --- | --- | --- |
|  | **RCY** | **RCP (HPLC)** | **RCP (TLC)** | **Mean peptide** | **Administered dose** |
| ***n. 1*** | 56.90% | 97.60% | 100% | 3.77 µg | 150 MBq |
| ***n. 2*** | 55.37% | 96.89% | 100% | 5.39 µg | 150 MBq |

**Table.** Radiosynthesis results of ^68^Ga-DOTATOC (a) and ^68^Ga-NODAGA-Exendin-4 (b), including radiochemical yield (RCY%), radiochemical purity (RCP%) assessed by HPLC and TLC, administered dose (MBq) and mean peptide amount (µg). *n.1* refers to the radiosyntheses carried out for the first PET scans with ^68^Ga-DOTATOC and ^68^Ga-NODAGA-Exendin-4 (performed in August 2023 and September 2023, respectively), while *n.2* refers to the radiosynthesis carried out for the second PET scans with the same tracers (October 2024 and November 2024, respectively)

**Figure.** (a) Radio-UV-HPLC chromatogram and TLC analysis of the first ^68^Ga-DOTATOC (August 2023); (b) Radio-UV-HPLC chromatogram and TLC analysis of the first ^68^Ga-NODAGA-Exendin-4 (September 2023).
